# Ectopic pleural thymoma with T-cell lymphocytosis and bone metastasis: a case report

**DOI:** 10.1186/s12890-024-03090-x

**Published:** 2024-06-14

**Authors:** Jun Li, Lisheng Liu, Jieping Li, Zailin Yang, Yao Liu

**Affiliations:** 1 Department of Hematology-Oncology, Chongqing University Cancer Hospital, No.181 HanYu Road, Chongqing, 400030 China; 2 Department of Nuclear Medicine, Chongqing University Cancer Hospital, Chongqing, 400030 China

**Keywords:** Ectopic pleural thymoma, T-cell lymphocytosis, Bone metastasis, Imaging features, Pathological features, Case report

## Abstract

**Background:**

The diagnostic complexities that arise in radiographic distinction between ectopic pleural thymoma and other thoracic neoplasms are substantial, with instances of co-occurring T-cell lymphocytosis and osseous metastasis being exceedingly rare.

**Case presentation:**

A 51-year-old woman was admitted to our hospital with dyspnea and chest pain. Upon imaging examination, she was found to have diffuse and nodular pleural thickening on the left side, collapse of the left lung and a compression in the second thoracic vertebrae. All lesions showed significant ^18^F-FDG uptake on ^18^F-FDG PET/CT examination. Furthermore, she exhibited T-cell lymphocytosis in her peripheral blood, lymph nodes, and bone marrow. After ruling out malignant pleural mesothelioma (MPM), lung cancer with pleural metastasis, and T-cell lymphoma, the definitive diagnosis asserted was ectopic pleural thymoma with T-cell lymphocytosis and bone metastasis.

**Conclusion:**

Physicians need to expand their knowledge of the imaging features of ectopic pleural thymoma. Cases with T-cell lymphocytosis may exhibit increased aggressiveness and prone to bone metastasis.

## Background

Ectopic pleural thymoma is a rare disease, with its radiographic characteristics often being challenging to differentiate from MPM and lung cancer accompanied by pleural metastasis. Due to the limited number of reported cases, the imaging features of ectopic pleural thymoma need to be further enriched. Notably, some literature suggests that thymomas associated with T-cell lymphocytosis exhibit increased invasiveness [1]. However, the rarity of such cases warrants further investigation. We report a rare case of ectopic pleural thymoma with T-cell lymphocytosis and bone metastasis, contributing to the deeper understanding of this condition.

## Case presentation

A 51-year-old woman was admitted to our hospital presenting with a two-month history of dry cough, dyspnea, and pain localized to the left chest and precordial region. The patient is a lifelong non-smoker and reported no infective, cardiac symptoms or myasthenia gravis (MG). Her weight was stable and there was no history of thoracic trauma or tuberculosis infection. Physical examination revealed rapid breathing for 28 breaths/min, decreased breath sound on the left chest and mild tenderness in the third thoracic vertebra region. No signs of hepatomegaly, splenomegaly, or skin lesions were observed.

Blood tests showed mild leukocytosis (13.30 × 10^9^/L) with lymphocytosis (9.98 × 10^9^/L). Absolutely CD4-positive T cell count and CD8-positive T cell count in the peripheral blood were 4,180 cell/ul and 1,679 cell/ul, respectively. Subsequent flow cytometric analysis confirmed that these lymphocytes were polyclonal T-cells. Her serum levels of procalcitonin (PCT) and C-reactive protein (CRP) were normal and no abnormalities in her liver and kidney functions. Ultrasound examination of the superficial lymph nodes revealed enlargement in the bilateral neck, axillary, and groin regions. Immunohistochemical staining of biopsy specimens from the cervical lymph nodes and bone marrow indicated CD3-positive, CD5-positive, CD7-positive small T-cell lymphocytosis. Moreover, gene rearrangement analysis did not detect any clonal rearrangement of the T-cell receptor (TCR). Flow cytometric examination of bone marrow showed mature T-cell lymphocytosis with normal phenotype.

**Fig. 1:**
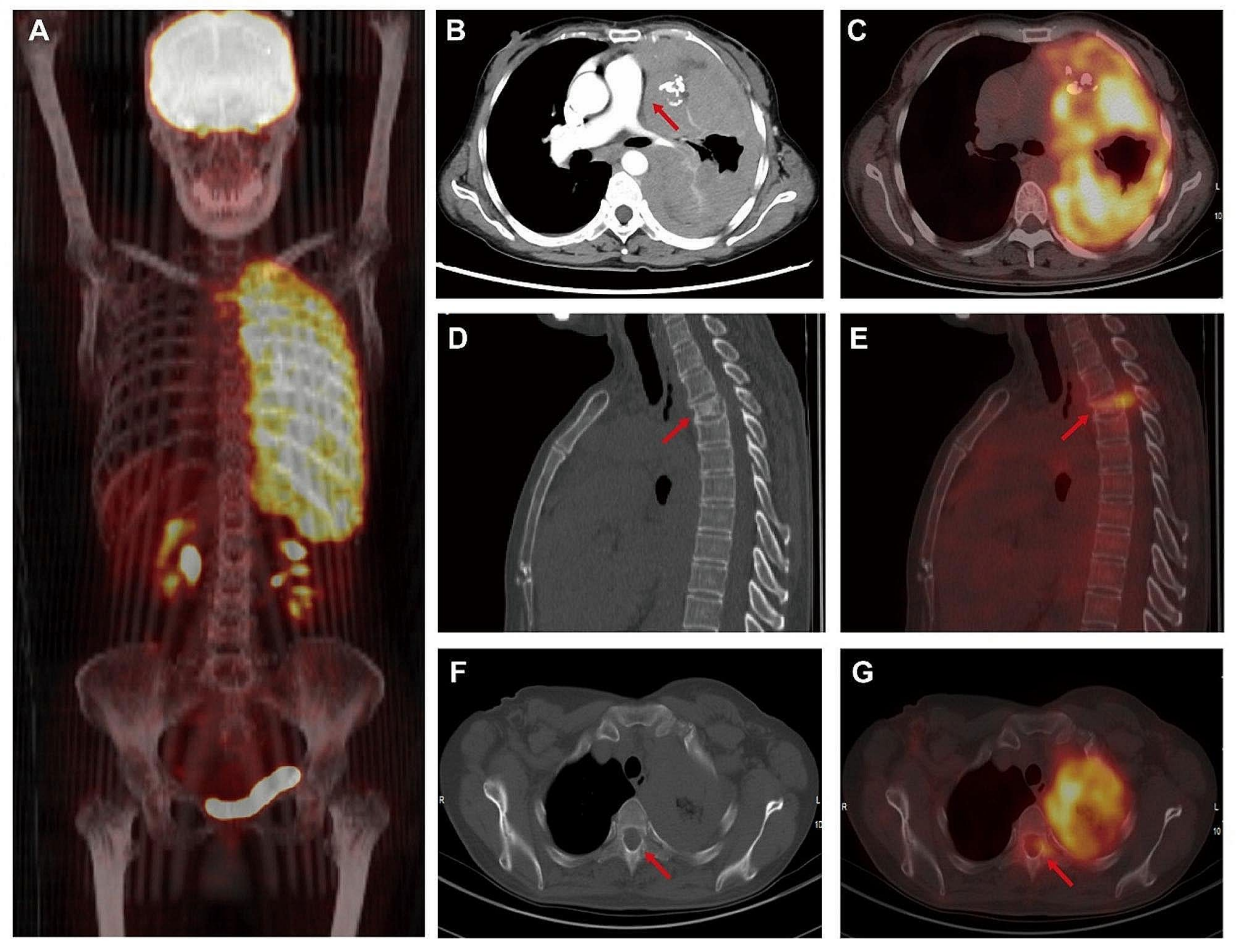
Radiographic imaging features of the chest. ^18^F-FDG PET/CT image showed increased ^18^F-FDG uptake of left pleura with a SUVmax of 8.3 (**A** and **C**). Contrast-enhanced CT image revealed the thickened pleura was mildly enhanced (**B**) and invading mediastinum and pericardium (arrow). A thoracic compression fracture was found in the second thoracic vertebrae (**D**, arrow) with a SUVmax of 8.2 (**E**, arrow). Osteolytic lesion of the second thoracic vertebrae attachment (**F**, arrow) was observed and it showed diffusely increased ^18^F-FDG uptake with a SUVmax 4.7 (**G**, arrow)

Contrast-enhanced chest CT scan depicted diffuse nodular thickening of the left pleura with mild enhancement, leading to the collapse of the left lung. This was seen infiltrating the mediastinum and pericardium (Fig. 1B). Bronchoscopy revealed stenosis of the left lower lobe bronchus, yet no malignant cells were detected in the bronchial mucosal biopsy specimens. The subsequent ^18^F-FDG PET/CT study indicated increased ^18^F-FDG uptake in the left pleura (SUVmax 8.3) (Fig. 1A, C). Additionally, compression with increased FDG uptake was observed in the second thoracic vertebrae (SUVmax 8.2) (Fig. 1D-G). Considering these findings, our differential diagnoses included malignant pleural mesothelioma, lung cancer with pleural metastasis, and T-cell lymphoma.

**Fig. 2:**
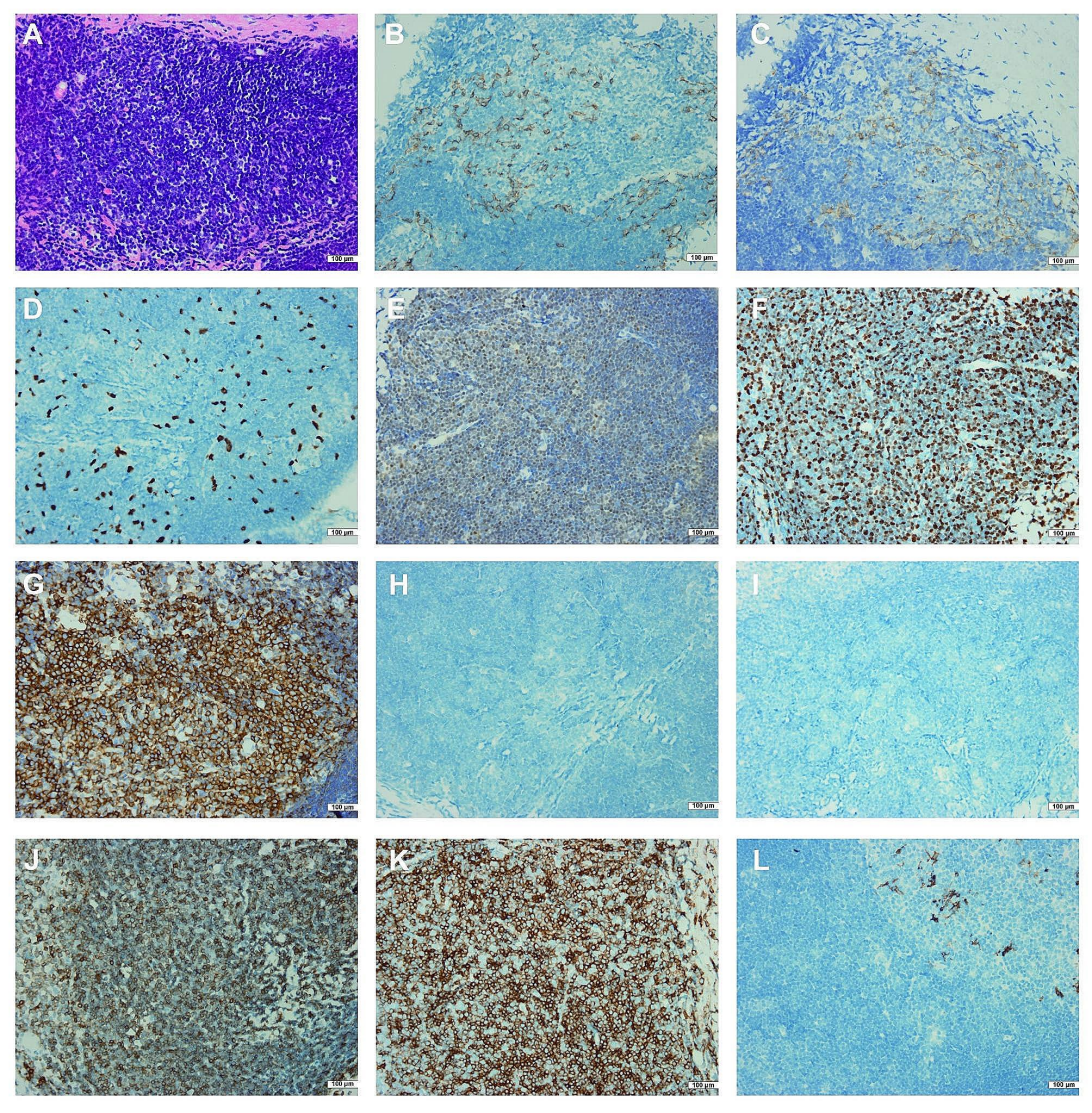
Histopathological fndings of pleural biopsy samples. Hematoxylin and eosin (H & E) stain revealed plenty of small-sized lymphocyte with scattered distribution of medium-sized cells (**A**). Immunohistochemical stain showed medium-sized cells were positive for PCK (**B**), CK19 (**C**), P63 (**D**), weak positive for TdT (**E**) and Ki-67 (**F**), but negative for CD5 (**G**), CR (**H**), and TTF-1 (**I**); while the small-sized lymphocytes were positive for CD3 (**J**), CD5(**G**), CD8(**K**) and Ki-67 (**F**), but negative for CD20 (**L**) .(**A**-**L**: original magnification×400)

Pleural biopsy revealed plenty of small-sized lymphocytes with scattered distribution of medium-sized cells in hematoxylin and eosin (H & E) staining (Fig. 2A). Immunohistochemical (IHC) stain showed medium-sized cells were positive for multiple epithelial-derived markers, including PCK (Fig. 2B), CK19 (Fig. 2C) and P63 (Fig. 2D). Additionally, these cells exhibited weak positivity for TdT (Fig. 2E) and Ki-67 (Fig. 2F), but were negative for CD5 (Fig. 2G), the mesothelial marker CR (Fig. 2H), and the pulmonary-origin marker TTF-1 (Fig. 2I). The small-sized lymphocytes were positive for CD3 (Fig. 2J), CD5 (Fig. 2G), CD8 (Fig. 2K) and Ki-67 (80%, Fig. 2F), but negative for CD20 (Fig. 2L) in IHC stain. Based on above findings, the patient was finally diagnosed as ectopic pleural thymoma (type B1 according to the WHO classification) with T-cell Lymphocytosis and bone metastasis. Regrettably, the patient declined further chemotherapy or radiotherapy options.

## Discussion and conclusion

In 2021, WHO updated the pathological types of thymoma, including type A, type AB and type B (B1, B2, B3) [2]. Among them, type B3 thymoma is considered to have a more aggressive clinical course and is more likely to metastasize [2]. However, factors predicting invasion and metastasis in some rare ectopic thymoma have not been fully explored. Ectopic thymoma is a rare condition that account only for 4% of all thymomas, which usually affect the neck, lung, pleura, pericardium, thyroid and middle/posterior mediastinum [3]. Ectopic pleural thymomas are frequently present as diffuse pleural tumors surrounding the lungs and mimicking MPM or metastatic lung cancer on radiological examinations [4]. Only a few cases have been reported about the peripheral T-cell lymphocytosis in thymoma, which seems to show a more aggressive clinical feature, but the underlying causes are not completely clear [5]. We report a rare case of ectopic pleural thymoma metastasized to the bone accompanied with T-cell lymphocytosis not only in the peripheral blood but also in lymph nodes and bone marrow. To our knowledge, this is the second case of ectopic thymoma with T-cell lymphocytosis and accompanying bone metastasis followed by Zhao et al. [5]. Our case have the following distinctive features.

Firstly, the patient had distinctive radiographic features when compared with the previously reported cases. In this case, diffused and nodular thickening of the left pleura with mildly enhanced in the contrast-enhanced CT and significantly elevated ^18^F-FDG uptake in the ^18^F-FDG PET/CT, occupying almost all the left thoracic cavity causing collapse of the left lung and invading mediastinum and pericardium, which was radiographically difficult to differentiate from MPN as well as from lung cancer with pleural metastasis [6]. Bouardi et al. reported a case of pleural thymoma characterized by a localized left pleural mass rather than diffuse involvement [7]. Shah et al. also reported a pleural thymoma presenting as a localized pleural-based mass with increased ^18^F-FDG uptake [8]. This indicates that it is difficult to diagnose ectopic pleural thymoma through imaging features, and pleural biopsy is necessary. Secondly, this case discovered a mature phenotype of T-cell lymphocytosis simultaneously involving in peripheral blood, enlarged lymph nodes and bone marrow in an ectopic pleural thymoma patient. Is this related to the bone metastasis of thymoma? To the date, many studies hold a view that peripheral T-cell lymphocytosis is an extremely rare paraneoplastic syndrome of thymoma and is regarded as a symbol of high invasive ability [1]. Zhao et al. summarized 11 cases from 1977 to 2013 and found that peripheral T-cell lymphocytosis was asscociated with aggressive thymomas in all these cases [5]. Thymic hormone released by malignant cells which mediate an immunoregulatory disorder might be the cause [5]. But the underlying mechanisms remain unclear and need to be further investigated.

In conclusion, this case highlights an impressive imaging features of ectopic pleural thymoma and a noval recognition of T-cell lymphocytosis in thymoma progression and metastasis to bone.

## Data Availability

Data are available from the corresponding author on reasonable request.

## Abbreviations

CD: Cluster of differentiation
CRP: C-reactive protein
FDG: Fluorodeoxyglucose
MG: Myasthenia gravis
MPM: Malignant pleural mesothelioma
PCT: Procalcitonin
PET/CT: Positron emission tomography/computed tomography
SUVmax: Maximum standardized uptake value
TCR: T-cell receptor
